# Repurposing of atorvastatin emulsomes as a topical antifungal agent

**DOI:** 10.1080/10717544.2022.2149898

**Published:** 2022-11-25

**Authors:** Alaa S. Eita, Amna M. A. Makky, Asem Anter, Islam A. Khalil

**Affiliations:** a Department of Pharmaceutics, College of Pharmaceutical Sciences and Drug Manufacturing, Misr University for Science and Technology (MUST), 6th of October, Giza, Egypt; b Department of Pharmaceutics and Industrial Pharmacy, Faculty of Pharmacy, Cairo University, Cairo, Egypt; c Microbiology Unit, Drug Factory, College of Pharmaceutical Sciences and Drug Manufacturing, Misr University for Science and Technology (MUST), 6th of October, Giza, Egypt

**Keywords:** Atorvastatin, emulsomes, repurposing, fungal infection, topical

## Abstract

Cutaneous fungal infection therapy confronts several issues concerning skin permeation in addition to drug resistance and adverse effects of conventional drugs. The repurposing strategy is supposed to overcome some of those therapeutic obstacles. Recently, atorvastatin (ATO) revealed antifungal activity. ATO is an antihyperlipidemic drug with pH-dependent solubility, which limits skin permeation. This study aims to improve ATO antifungal activity by encapsulation in an emulsomes (EMLs) system, which seeks to ameliorate skin penetration. Therefore, multiple factors were investigated according to the One-Factor-at-a-Time (OFAT) design to achieve the optimum formula with targeted characteristics. Minimizing particle-size and polydispersity-index, besides elevating zeta-potential (ZP) and entrapment-efficiency were the desirable responses during assessing 11 factors. The selected ATO-EMLs formula (E21) recorded 250.5 nm in particle size, polydispersity index of 0.4, ZP of –25.93 mV, and 83.12% of drug entrapped. Morphological study of E21 revealed spherical core–shell vesicles in nanosize. DSC, XRD, and FTIR were conducted to discover the physicochemical properties and confirm emulsomes formation. Optimized ATO-EMLs slowed drug release rate as only 75% of ATO was released after 72 h. Stability study recommended storage between 2 and 8 °C. The *in vivo* permeation study remarked a homogeneous penetration of EMLs in different skin layers. The *in vivo* skin irritation test revealed limited histopathological changes. The *in vitro* and *in vivo* microbiological studies demonstrated a good antifungal activity of ATO-EMLs. ATO-EMLs system improved antifungal activity as the MIC values reduced from 650 µg/mL for free ATO to 550 µg/mL for ATO-EMLs. These findings may shed light on ATO as an antifungal drug and nanosystems as a tool to support drug repurposing.

## 1. Introduction

Fungal infections are one of the highly insecure health issues faced by healthcare professionals. Statistics recorded about 13 million infections and ∼1.5 million deaths worldwide yearly. One of the main causes of this alarming rate is the noticeable elevation in the immunocompromised patient population (Rayens & Norris, 2022). Candidiasis is one of the most prevalent diseases affecting patients globally, including various types such as mucosal candidiasis, systemic candidiasis, vaginal candidiasis, and cutaneous candidiasis (Kauffman et al., 2011). Cutaneous candidiasis can be treated with several antifungal classes such as azole, allylamine, and polyene antifungals. Nowadays drug resistance and adverse effects are revealed as an obvious challenge for those conventional antifungal drugs. Therefore, prospective studies and drug development are in demand (de Oliveira Santos et al., 2018).

In the pharmaceutical industry, establishing a new drug is a complicated, costly, and time-consuming process. According to the food and drug administration (FDA), the drug development process generally undergoes four stages before marketing including discovery and development, preclinical, and clinical research. FDA reviews all those stages over 12–15 years (Hughes et al., 2011). A new strategy termed drug repurposing ‘repositioning’ was useful to overcome the lengthy drug discovery process. Repurposing implies the ability to investigate new therapeutic uses for previously approved drugs with different utilization scopes (Pushpakom et al., 2019). Recently, this strategy is widely used effectively in oncology, cardiology, mycology, and other diseases (Peyclit et al., 2021). Many drug classes were investigated for antifungal efficacy, and chosen as useful alternative common drugs (Miró-Canturri et al., 2019; Kim et al., 2020).

Statins are a recognized group of drugs that are primarily used as plasma cholesterol-lowering agents upon inhibiting the hydroxy-methyl-glutaryl-CoA (HMG-CoA) reductase enzyme (Sirtori, 2014). New studies on statins realized new therapeutic fields such as cancer therapy (Jiang et al., 2021), antibacterial (Ko et al., 2018), and antifungal treatment. Tavakkoli et al. showed the antifungal effects of statins against wide pathogenic species and reported that the HMG-CoA reductase enzyme was also revealed as a fungal enzyme; so, it will expose to inhibition by statins, which leads to fungal growth suppression due to ergosterol level decline (Tavakkoli et al., 2020). Atorvastatin (ATO) was reported as one of the statin categories effectively involved in antifungal therapeutic studies as it was investigated previously against *Candida albicans* (Nasr Esfahani et al., 2019). ATO-loaded solid lipid nanoparticles were reported to promote drug permeation through skin layers for anti-inflammatory activity (Shahraeini et al., 2020).

Vesicle nanocarriers possess distinctive features as topical drug delivery. Vesicle size allows drug penetration through skin layers and provides a controlled drug release (Sinico & Fadda, 2009). Liposomes are the first established system that was developed for topical and transdermal drug delivery. However, different modifications were investigated to improve its stability and delivery efficiency. Over the past decade, several generations of vesicle systems were reported such as niosomes, transfersomes, and emulsomes (Alhakamy et al., 2020). The emulsome system acquired a favorable environment for loading lipophilic drugs as it consists of a lipid core surrounded by a phospholipid (PL) bilayer that can retain higher lipophilic drug amounts. Moreover, water-soluble drugs can be encapsulated in the aqueous site of the outer shell layer (Bolat et al., 2020). Subsequently, emulsome is noticed as a reliable drug delivery system due to proper drug entrapment efficiency, biodegradability, biocompatibility, and controlled drug release (Zhou & Chen, 2015).

**Scheme 1.**
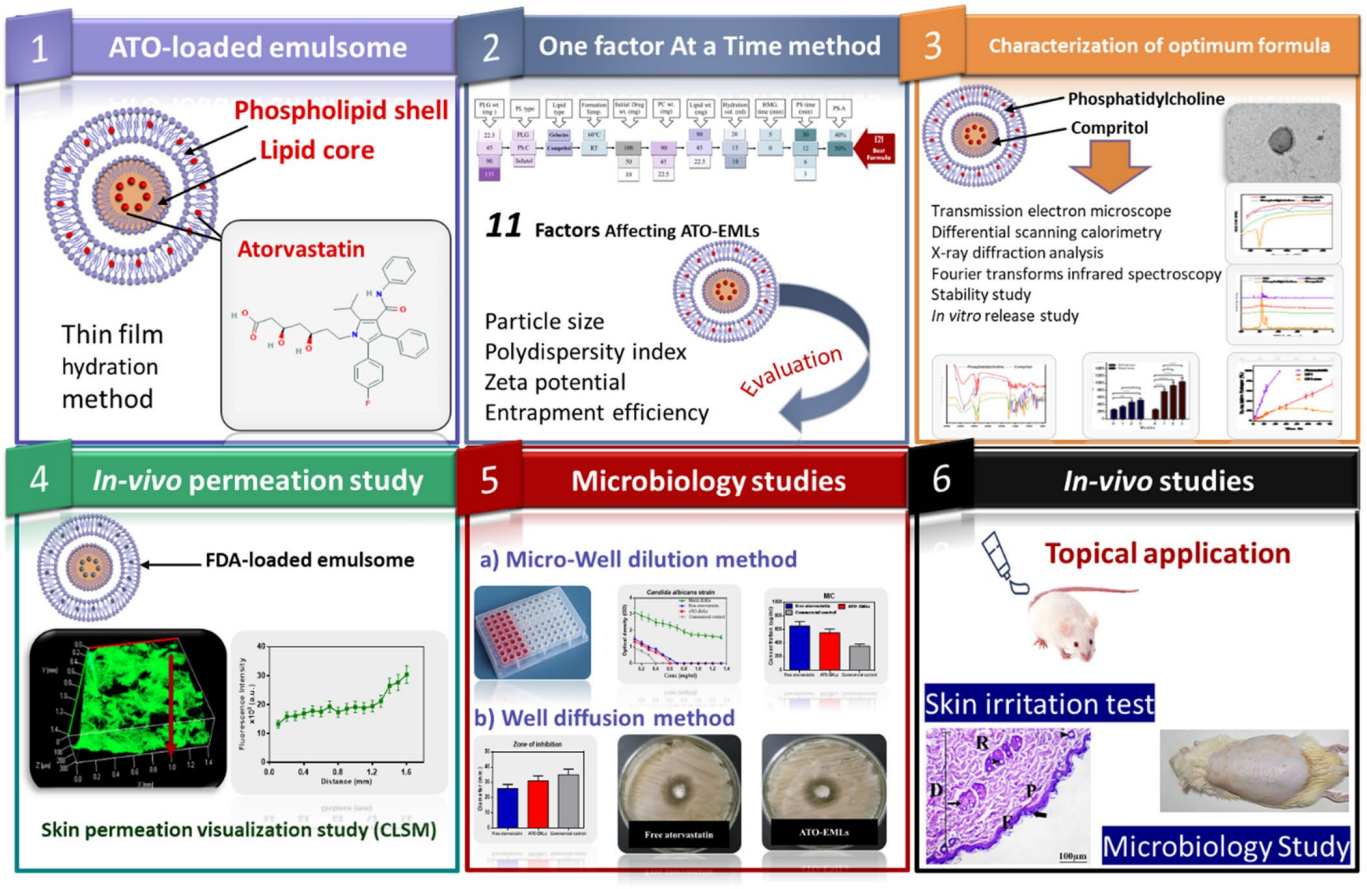
Study design**:** Phase 1 represents the formulation of atorvastatin-loaded emulsome, Phase 2 illustrates studying factors affecting ATO-EMLs using OFAT design and evaluating formulae for the best selection, Phase 3 shows further characterization for optimum formula, Phase 4 proves skin permeation by *in vivo* visualization study, Phase 5 represents microbiology studies to evaluate the antifungal activity of atorvastatin, Phase 6 assigned for the *in vivo* studies.

This study aims to improve ATO potential as an antifungal drug by encapsulation into an emulsome (EML) vesicle system. EMLs were selected to allow deep penetration into the skin layers and improve drug activity. ATO-loaded EMLs were optimized by studying different factors. The optimum formula was subjected to *in vivo* permeability and skin irritation studies besides *in vitro* and *in vivo* microbiology studies. These studies were conducted to estimate the effectiveness of ATO against deep skin fungus infection. The study design is summarized in Scheme 1.

## 2. Materials and methods

### 2.1. Materials

Atorvastatin calcium was kindly gifted by EIPICO, Egypt. Phospholipon® 90G was a gift sample from Phospholipid GmbH (Nattermannallee, Germany). Soya phosphatidylcholine, Solutol, and cellulose membrane (Molecular weight cut off: 12,000) were purchased from Sigma-Aldrich (St. Loius, MO, USA). Compritol 888 ATO and Gelucire 40/14 (PEG glyceride) were a kind gift from Gatteffose, France. Potassium dihydrogen phosphate and di-sodium hydrogen phosphate were purchased from El-Nasr Pharmaceutical Co. (Cairo, Egypt). All other used chemicals were of analytical grade.

### 2.2. Preparation of ATO-EMLs

ATO-EMLs were prepared as per the reported method (El-Zaafarany et al., 2016; Sawant et al., 2016) using a thin-film hydration technique with slight modification. ATO was solubilized first in a sufficient methanol volume, while specified quantities of other components were solubilized in a mixture of chloroform/methanol (2:1, vol/vol) before mixing. Using rotary evaporator under reduced pressure until complete solvent evaporation occurred. The residual films were then hydrated with a specified volume of deionized water with gentle agitation for 1 h, and the resulting formulae were subjected to bath sonication for 1 min and then probe sonication under variable conditions (750 W, 20 kHz).

### 2.3. Experimental design

The structure of the study was formed according to the One-Factor-at-a-Time (OFAT) design. Different variables were studied gradually by changing only one factor at each trial and keeping the rest fixed. Eleven factors have been investigated as shown in Table 1: PL amount expressed as phospholipon G (PLG) (22.5, 45, 90, 135 mg), types of PL (PLG, phosphatidylcholine (PC), and solutol), lipid types (Compritol and Gelucire), the temperature during emulsome formation (60 °C, and room temperature(RT)), initial drug amount (10, 50, and 100 mg), PC amount (22.5, 45, and 90 mg), lipid amount (22.5, 45, and 90 mg), hydration volume (10, 15, and 20 mL), homogenization effect, probe sonication time (3, 6, 12, and 30 min), and probe amplitude (40%, and 50%).

**Table 1.** ATO-EMLs formulation according to the One Factor at a Time (OFAT) method.

F No.	PL wt. (mg)	PL type	Lipid wt. (mg)	Lipid type	Drug wt. (mg)	Hydration volume (ml)	EMLs. formation temp. (°C)	HMG. duration (min)	PS duration (min)	PS amplitude (%)
E1	22.5	PLG	45	Comp.	10	10	60	0	3	40%
E2	45	PLG	45	Comp.	10	10	60	0	3	40%
E3	90	PLG	45	Comp.	10	10	60	0	3	40%
E4	135	PLG	45	Comp.	10	10	60	0	3	40%
E5	45	PC	45	Comp.	10	10	60	0	3	40%
E6	45	Solu.	45	Comp.	10	10	60	0	3	40%
E7	45	PC	45	Geluc.	10	10	60	0	3	40%
E8	45	PC	45	Comp.	10	10	RT	0	3	40%
E9	45	PC	45	Comp.	50	10	RT	0	3	40%
E10	45	PC	45	Comp.	100	10	RT	0	3	40%
E11	22.5	PC	45	Comp.	100	10	RT	0	3	40%
E12	90	PC	45	Comp.	100	10	RT	0	3	40%
E13	90	PC	22.5	Comp	100	10	RT	0	3	40%
E14	90	PC	90	Comp.	100	10	RT	0	3	40%
E15	90	PC	45	Comp.	100	15	RT	0	3	40%
E16	90	PC	45	Comp.	100	20	RT	0	3	40%
E17	90	PC	45	Comp.	100	15	RT	5	3	40%
E18	90	PC	45	Comp.	100	15	RT	0	6	40%
E19	90	PC	45	Comp.	100	15	RT	0	12	40%
E20	90	PC	45	Comp.	100	15	RT	0	30	40%
E21	90	PC	45	Comp.	100	15	RT	0	12	50%

PL, Phospholipid; wt., weight; Temp., Temperature; HMG., Homogenization; PS, Probe sonication; PLG, Phospholipon G; PC, Phosphatidylcholine; Solu., Solutol; Comp., Compritol; Geluc., Gelucire; RT, room temperature.

Studied different factors aiming to optimize a vesicle system that possesses the minimum particle size (PS) in an acceptable range with a minimum polydispersity index (PDI), higher zeta potential (ZP) as an absolute value, and maximum entrapment efficiency percent (EE%).

### 2.4. Evaluation of ATO-EMLs

#### 2.4.1. Dynamic light scattering

The dynamic light scattering (DLS) technique was used at 25 °C to assess PS, PDI, and ZP for various emulsome formulae using Zetasizer (Malvern Instruments, Malvern, UK). Deionized water was used to dilute the nanovesicle dispersion. Means and standard deviations for triplicate measurements were computed.

#### 2.4.2. Entrapment efficiency

Atorvastatin EE% in the emulsomes system was measured indirectly. One mL of ATO-loaded emulsomal dispersion system (amounting to 6.7 mg ATO) was centrifuged at 20,000 rpm for 1 h at 4 °C using a cooling centrifuge (Sigma 3K 30, Germany) to separate the unentrapped drug. The residue was washed twice with deionized water and then recentrifuged for 1 h as the method reported by Aldawsari et al. (2021) and Sawant et al. (2016) with slight modulation. The combined supernatant was analyzed using UV–vis spectrophotometer at 238.6 nm (Shimadzu UV 1650 Spectrophotometer, Japan). All determinations were made in triplicate. EE% was determined using the following equation:

$$\mathrm{EE}\%=\frac{\mathrm{TD}-\mathrm{UD}}{\mathrm{TD}}\times 100$$

where TD and UD denote the amount of total drug and unentrapped drug in the supernatant, respectively.

### 2.5. Characterization of optimum formula

#### 2.5.1. Vesicle morphology

A transmission electron microscope (TEM) was utilized to inspect ATO-EMLs morphology (TEM; CM12; Philips, USA). The investigation focused on the best vesicle formulation. To visualize the emulsome vesicle structure, the sample was dried on a carbon-coated grid and stained negatively with an aqueous solution of 1% phosphotungstic acid. The sample was imaged using TEM after the stain had dried (Elbaz et al., 2016).

#### 2.5.2. Differential scanning calorimetry

Thermal analysis was achieved using a differential scanning calorimeter (DSC, Shimadzu TA-60, Japan). Analysis was performed on free ATO, Compritol, PC, and ATO-EMLs vesicles. Each sample was scanned at temperatures ranging from room temperature to 300 °C. Thermograms were obtained at a scanning rate of 10 °C/min (Abdellatif et al., 2017).

#### 2.5.3. X-ray diffraction analysis

Using X-ray diffraction (XRD) analysis revealed diffraction patterns of ATO pure powder, Compritol, PC, and optimized lyophilized ATO-loaded emulsome formulation. The diffractograms in a Scintag X-ray diffractometer (USA) were recorded at 45 kV, the current 30 mA, the steps 0.02, and the counting rate of 0.5 s/step at room temperature (Nasr et al., 2015; Shamma et al., 2019).

#### 2.5.4. Fourier-transform infrared spectroscopy

Fourier-transform infrared (FTIR) spectroscopy studies were applied on free ATO, PC, Compritol, and optimum formula using a spectrophotometer (Shimadzu IR- Affinity-1, Japan). The scanning was applied in the range of 399–4000 cm^−1^ at a resolution of 4 cm^−1^ at ambient temperature with a speed of 2 mm/s (Zhao et al., 2015).

### 2.6. Stability study

Attributes of optimized formulation during storage in a glass vial at 4 ± 1 °C and 25 ± 1 °C under controlled humidity of 75% RH have been studied. Physical appearance, PS, PDI, ZP, and EE% were evaluated to reflect the physicochemical stability of prepared ATO-EMLs. Measurements were taken immediately in triplicate after the vesicle system was prepared and at various time intervals over three months.

### 2.7. *In vitro* release study

The release study was carried out using a diffusion technique with a surface area equal to 5 cm^2^. A 100 mL of the diffusion media consisting of PBS (pH: 5.5) containing 10% propylene glycol to maintain sink condition was kept in the receiver chamber at 32 °C by continuous stirring at 100 rpm, 1.5 mL of the emulsomal suspension containing a fixed weight of ATO (10 mg) was placed in the donor compartment of the diffusion cell. At predetermined time intervals, samples were withdrawn, and the drug concentration was determined using a UV/visible spectrophotometer. The receptor chamber was then compensated with equal volumes of fresh medium.

Core drug entrapment was determined by adding excess organic solvent (ethanol) to emulsomal suspension to ensure the complete dissolution of the shell layer and the drug entrapped in it. Subsequently, a cooling centrifuge was used to separate the dissolved drug and PL components in the supernatant from the drug which was still entrapped in the pellet. Later, the pellet was redispersed in deionized water, and the amount of drug entrapped in the lipid core was determined using a diffusion cell under the same condition as the previous release study.

Data obtained from the release study was subjected to zero order, first order, second order, Weibull model, Higuchi model, and Hixson–Crowell model, etc., looking for the best fitting one with a higher correlation coefficient (*R*^2^) using (KinetDS) software (Mendyk et al., 2012) and data interpretation according to Costa and Sousa Lobo and Zhang et al. (Costa & Sousa Lobo, 2001; Y. Zhang et al., 2010).

### 2.8. *In vivo* permeation visualization study

Depth of skin penetration of the vesicle system was simulated by loading a fluorescent dye in the formula that was inspected using confocal laser scanning microscopy (CLSM), as targeting deep layers of the skin was desired (Alvarez-Román et al., 2004).

#### 2.8.1. Preparation of FDA-loaded emulsomal dispersion

Emulsomes loaded with fluorescein diacetate (FDA) were prepared by using the thin-film hydration technique as mentioned previously, while FDA was added instead of ATO.

#### 2.8.2. Skin preparation

The *in vivo* permeation study was carried out according to the guidelines approved by the ethical committee of the Department of Pharmacy, Cairo University (serial number of the protocol PI 2850). An albino rat was prepared by removing the hair after drawing a specific mark on the dorsal area about approximately 2 cm^2^. FDA-loaded nanovesicle was applied to the identified area and remained for 24 h. After application, the rat was sacrificed by a cervical dislocation method, and skin tissue including the application zone was excised and kept in the freezer (–20 °C) until CLSM investigation, as reported in Kassem et al. where utilizing *in vivo* visualization strategy using CLSM for vesicle formulation (niosomes) on rat skin (Kassem et al., 2017).

#### 2.8.3. Confocal laser scanning microscopy study

The longitudinal section was investigated for fluorescence in the skin tissues upon being placed between a glass slide and a cover slip. The slides were visualized using a microscope (LSM 710; Carl Zeiss, Oberkochen, Germany). The excitation and emission wavelengths of the FDA were *λ*_max_ 488 nm and *λ*_max_ 530 nm, respectively. The skin thickness was optically scanned at 15 µm increments from the skin surface of 0 µm to a depth of 255 µm. The penetration of the emulsome system in different skin layers was reflected by tiling and stitching several individual *x*-*y* images. Moreover, fluorescence intensity profile across skin layers was measured to detect the disposition of emulsomes. Finally, structuring a 3D plot using Z-stack mode was conducted as previously reported (Shamma & Aburahma, 2014).

### 2.9. *In vitro* microbiological study

#### 2.9.1. Fungal strains

Standard strains of *C. albicans* employed in this work were obtained from the culture collection unit at the Regional Center for Mycology and Biotechnology (Al Azhar University). Each strain was sub-cultured on sabouraud dextrose agar (SDA) medium and incubated at 37 °C for 24 h to obtain inoculums for testing.

#### 2.9.2. Preparation of the inoculums

About two to three colonies from 24-h old culture were used as the inoculum following suspension in 10 mL of 0.85% sodium chloride solution, which was autoclaved. The turbidity was adjusted to 0.5 McFarland standard units (i.e. 1.5 × 10^8^ CFU/ml) (Alyousef, 2021).

#### 2.9.3. Micro-dilution method

The micro-dilution method was used to determine the minimal concentration of free ATO suspension, ATO-EMLs, and blank EMLs formulae that induced the inhibition of visible yeast growth or turbidity, which is called minimum inhibitory concentration (MIC) (Rodríguez-Tudela et al., 2003). Twofold serial dilutions of the extracts were inoculated directly in a microtiter plate containing Sabouraud broth to obtain concentrations ranging from 1.32 to 0.1 mg/mL. Subsequently, each well was added to a final volume of 100 μL of the strain inoculum (adjusted according to 0.5 McFarland standard units). The experiment was performed in triplicate. The culture plates were incubated at 37 °C for 24 h overnight. Negative control (Sabouraud broth only) and positive controls (Sabouraud broth and microorganism) were tested to determine medium sterility and inoculum viability, respectively. The activity of Fungistate (1% gel) as commercial control was also assessed with formulae dilutions as a standard. The lowest concentration at which there was no turbidity was determined as the MIC of the formulae.

#### 2.9.4. Well diffusion method

Anticandidal activity using a well diffusion assay of free ATO suspension, ATO-EMLs, and blank EMLs formulae was conducted. First, SDA media was prepared and autoclaved. About 15–20 mL of the media was poured into sterile Petri dishes, spread homogeneously, and remained solidified for 30 min. Thereafter, 0.2 mL of inoculum (fungal strain in saline) was spread on an agar plate, and the excess was discarded via draining. The plates were then incubated at ambient temperature for 10 min. Subsequently, a ditch of 4 mm was shaped in each plate, using a sterile cork borer. Each well was filled with 50–100 μL of the diluted formulae. Commercial Fungistate 1% gel was used as positive control while saline was used as a negative control. The plates were incubated for 24 h at 37 °C. The zone of inhibition was measured in millimeters (Senyiğit et al., 2014). The assay was triplicated for confirmation.

### 2.10. *In vivo* studies

#### 2.10.1. Skin irritation test

*In vivo* skin irritation test was conducted to assess the safety of the developed emulosmes formula. This study was carried out according to the guidelines approved by the ethical committee of the Department of Pharmacy, Cairo University (serial number of the protocol PI 2850). Twelve male Wistar albino rats (200–250 g) were assigned to two groups (6 rats for each group). Group I was assigned to the placebo emulsomes formula and Group II was assigned to the ATO-loaded emulsomes formula. All rats had access to normal chow and ad Lib. The hair of the dorsal side of the rats was shaved followed by the application of the formula. The skin irritation and erythema were observed for 3 weeks to assess the safety of the formulations. Rats were sacrificed at two-time points (one and three weeks) by the cervical dislocation method. Skin specimens were obtained from rat dorsum, followed by washing with normal saline and fixed in 10% buffered formalin solution for 24 h, then trimmed off, washed, dehydrated, cleaned, and embedded in paraffin. Sequential skin sections of 5–7 μm thickness were cut and then mounted on glass slides. The obtained tissue sections were deparaffinized using xylol and stained using hematoxylin and eosin (H&E) for histopathological examination through the electric light microscope (Suvarna et al., 2012). The histopathological findings were evaluated as scores graded as follows: – (no abnormality), + (slight), ++ (mild), +++ (moderate) for changes as previously reported (Watanabe et al., 2009).

#### 2.10.2. Microbiological study

*In vivo* microbiological study was conducted to assess the antimicrobial activity of the developed emulosmes formula. This study was carried out according to the guidelines approved by the ethical committee of the Department of Pharmacy, Cairo University (serial number of the protocol PI 2850). Eighteen male Wistar albino rats (200–250 g) were assigned to three groups (6 rats for each group). Group I was assigned to the positive control (skin infection without treatment). Group II was assigned to the standard antifungal drug (Terbinafine HCl) with a once-daily dose. Group III was assigned to the ATO-loaded emulsomes formula with one dose every 3 days. For animal preparation, rats were housed individually with free access to food and water. Subcutaneous injection of betamethasone (2 mg/kg body weight/day) three times daily for three days was administered to suppress rat immunity followed by 100 µL of 10^7^ CFU/mL intradermally injection of *C. albicans* (ATCC 10231) to the shaved dorsal skin of each rat (Abdellatif et al., 2017). Signs of fungal skin infection were observed 3 days after fungal induction. The treatment was continued for three weeks followed by scarification by the cervical dislocation method. The clinical symptoms of fungal skin infection were observed such as skin erythema. Skin specimens were collected from each group for histopathological and microbiological evaluations. For microbiological evaluation, skin specimens were washed, homogenated, and incubated in sabourad dextrose agar culture media for 48 h at 37 °C. The colony-forming units (CFU) were counted and recorded. Histopathological examination was conducted as previously mentioned in the skin irritation test.

### 2.11. Statistical analysis of data

Student’s *t* test and one-way analysis of variance (ANOVA) with Tukey’s post hoc tests were used to determine the significant difference between the formulae under investigation. Significance level set at 0.05, and (*p < .05, **p < .01, ***p < .001, and ****p < .0001) assumed to be statistically significant by using Graph Pad Prism software (Version 6). All data were presented as means ± standard deviation (*n* = 3).

## 3. Results and discussion

Topical drug delivery mainly faces many changes due to the unique structure of the skin. The nature of the skin layers creates an obstacle to the penetration of topical antimicrobial drugs and hinders their activity. Furthermore, the physicochemical properties of drugs limit their availability at a therapeutic level that leads to drug resistance. Therefore, discovering and repurposing new drugs were widely investigated. Nowadays, new studies generally focus on encapsulation in nanosystems with specific characterization to overcome those limitations. For this reason, this study focuses on developing emulsomes as a promising system for topical delivery of Atorvastatin (ATO-EMLs) as a repurposed drug for dermal fungal infection. Atorvastatin calcium has a pH-dependent solubility (p*K*_a_ = 4.46 and log*P* = 6.36), which limits its penetration through skin layers. To achieve suitable topical delivery, the nanosystem was optimized to overcome Atorvastatin calcium physicochemical properties and target dermal fungal infection.

### 3.1. Evaluation of ATO-EMLs

As shown in Table 1, according to the OFAT method, the effects of several factors were evaluated stepwise, and the results were analyzed to achieve the best formulae with the predetermined characteristic, which are minimum PS and PDI beside maximum drug entrapment and surface charge (absolute).

#### 3.2.1. PL amount

**Figure 1.**
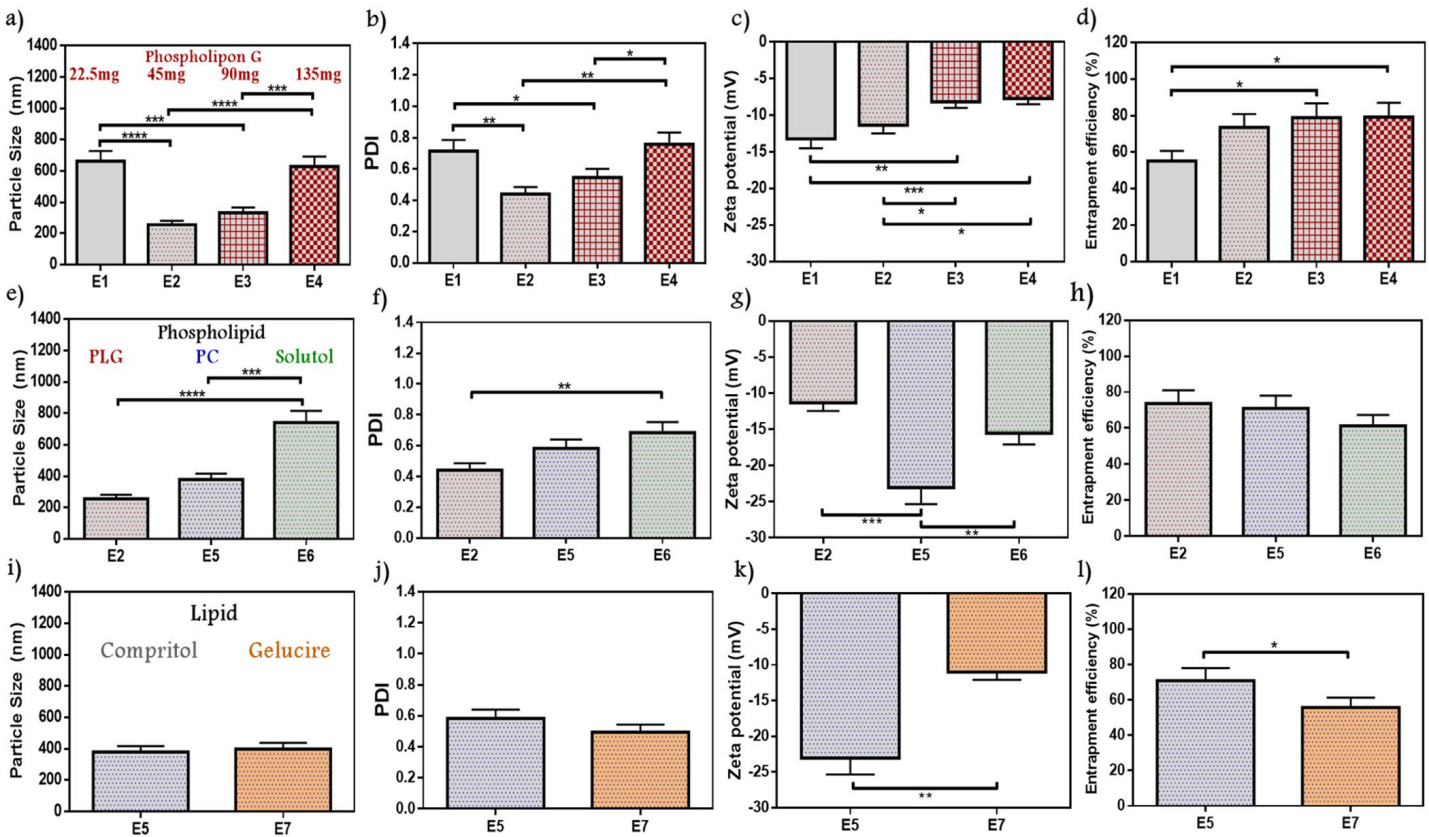
Emulsome evaluation: factor 1; effect of phospholipon G on (a) particle size, (b) polydispersity index, (c) zeta potential, and (d) entrapment efficiency percent; E2 was selected according to particle size, polydispersity index, and zeta potential, factor 2; the effect of phospholipid types on (e) particle size, (f) polydispersity index, (g) zeta potential, and (h) entrapment efficiency percent; E5 was selected according to particle size, and zeta potential, factor 3; the effect of lipid types on (i) particle size, (j) polydispersity index, (k) zeta potential, and (l) entrapment efficiency percent; E5 was selected according to zeta potential, and entrapment efficiency. Data are represented as mean ± SD (*n* = 3; *p < .05, **p < .01, ***p < .001 and ****p < .0001).

In formulae from E1 to E4, fixed Compritol amounts (45 mg) were used with different amounts of PLG while all other variables were constant. As shown in Figure 1(a,b), all vesicles size were on the nanoscale, ranging from 254.7 to 661.8 nm, and PDI values were between 0.44 and 0.76. E2 in comparison with E1 and E4 showed significantly decreased in PS (*p* < .0001) and PDI (*p* < .01). Although all formulations exhibited negative ZP (–7.7 to –13.2 mV), as shown in Figure 1(c). E2 appeared significantly higher than E3 and E4 (*p* < .05). Entrapment efficiency values measured for E2 to E4 formulae were in the range of 73.62 to 79.22%, while E1 showed only 55.23% of drug entrapped (Figure 1(d)). As a result, E2 which contains 45 mg of PLG possesses the lower PS and PDI (254.7 nm and 0.44, respectively), with acceptable ZP (–11.34 mV) and EE% (73.62%) values. Sawant et al. reported using an emulsome system for loaded sparfloxacin at different PLG and Compritol amounts, where a decrease in PS results by increasing PLG up to nearly 50% of total lipid amount, otherwise further increase led to counter effect. It is remarked that exaggerated PLG concentration may initiate liposome formation with higher PS. Furthermore, it noticed an increase in EE% with higher PL amount which may be related to drug lipophilicity, this point of view agrees with our result (Sawant et al., 2016). E2 was selected as minimizing PS improved vesicle penetration through stratum layers of the skin (Mosallam et al., 2021).

#### 3.2.2. PL type

A weight of 45 mg of PLG in E2 formulae was selected to be compared with other PL types; PC in E5, and solutol in E6 (Figure 1(e–h)). The PS showed a higher increase with solutol (740.6 nm) than PC (378 nm) and PLG, a remarked significant decrease in the size of E5 than E6 was obtained (*p* < .001). The PDI of E5 and E6 were 0.58 and 0.68, respectively. E5 represented significantly higher ZP (–23.03 mV) than E6 (–15.52 mV) (*p* < .01) as well as E2 (–11.34 mV) (*p* < .001). A slight decrease in entrapment appeared in the case of solutol as only 61.22% was entrapped instead of 73.62%, and 70.92% in the case of PLG and PC, respectively. E5 was selected according to maximized ZP for studying subsequent variables. As reported by Zhang et al. while targeting dermal fungus infection using vesicle nanocarrier loaded with terbinafine hydrochloride, a maximizing in ZP contributed to higher repulsive forces that enhanced vesicle stability and improved the interaction with the skin layers, which is in agreement with current findings (L. Zhang et al., 2020).

#### 3.2.3. Lipid type

The lipid type factor was conducted by comparing Gelucire as a lipid core in formula E7 instead of Compritol in E5 while all other variables were constant. A close value of PS for the two lipids has been noticed in Figure 1(i) (378 nm for E5, and 397.3 nm for E7), while PDI were 0.58 and 0.49, respectively for E5 and E7 (Figure 1(j)). As shown in Figure 1(k,I), gelucire formula owns a significantly lower ZP (–10.98 mV) when compared with the Compritol formula (*p* < .01). Compritol formula E5 showed significantly higher drug entrapment of 70%, while only 55.75% was entrapped for gelucire core E7 (*p* < .05). Accordingly, Compritol lipid core (E5) showed more convenient properties, so it was selected as the lipid type of choice. El-Zaafarany et al. reported during the evaluation of different lipid types among studying oxcarbazepine-loaded emulsome that Compritol remarked preferable consistency with PC and better vesicle assembly (El-Zaafarany et al., 2016). Furthermore, Aburahma and Badr-Eldin mentioned that Compritol acquired a higher entrapment capacity for the lipophilic drug due to longer hydrocarbon chain length that resulted in interchain intercalation and promote intermolecular entrapment, those results supported our selection in lipid type factor (Aburahma & Badr-Eldin, 2014).

#### 3.2.4. Temperature during emulsome formation

**Figure 2.**
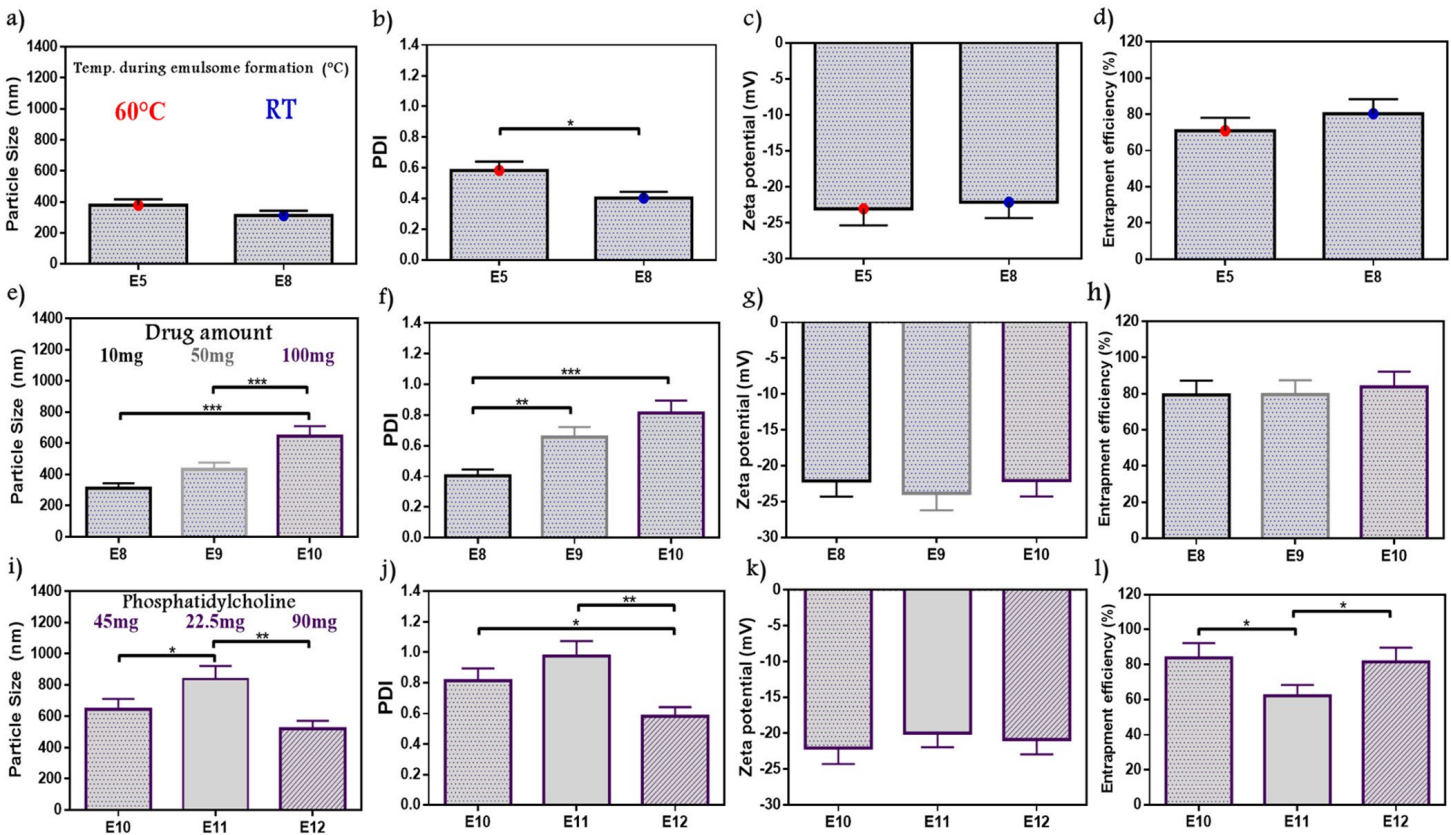
Emulsome evaluation: factor 4; effect of the temperature applied during emulsome hydration on (a) particle size, (b) polydispersity index, (c) zeta potential, and (d) entrapment efficiency percent; E8 was selected according to polydispersity index, factor 5; the effect of initial drug amount on (e) particle size, (f) polydispersity index, (g) zeta potential, and (h) entrapment efficiency percent; E10 was selected according to the study dose requirement, factor 6; the effect of phosphatidylcholine amount on (i) particle size, (j) polydispersity index, (k) zeta potential, and (l) entrapment efficiency percent; E12 was selected according to particle size, polydispersity index, and entrapment efficiency. Data are represented as mean ± SD (*n* = 3; *p < .05, **p < .01, and ***p < .001).

In a way to determine the favorable conditions for ATO-EMLs formation, the rotation temperature during vesicle formation was studied as shown in Figure 2(a–d). E5 formula has been formed at about 60 °C, while a new formula with the same variables was prepared and rotated at room temperature (E8). No significant differences have been observed in PS whereas the new *z*-average value was 312 nm, but the PDI was remarked to be preferable as it was about 0.4 which reflects a significant decrease in E8 than E5 (*p* < .05). The ZP of E8 was –22.11, and the amount of drug entrapped rise to 80.3%. E8 that rotated at room temperature showed lower PDI, and the visual appearance of the forming film reflected higher stability and better homogeneity. Kumar et al. upon studying emulsomes vesicle system formulation elements and preparation methods noticed that lipid core materials required room temperature (25 °C) for solidification and stabilization, that concept supported E8 selection (Kumar et al., 2013).

#### 3.2.5. Initial drug amount

According to the study objectives, a higher drug amount was desired for easier application, therefore the initial drug amount (10–100 mg) was evaluated to decide whether it was acceptable for the system to fit with the elevated drug amount or not. Variations in PS and PDI were observed in Figure 2(e,f), the PS values were in the range of 312–645.2 nm with a significant increase in E10 than E8 and E9 (*p* < .001), while PDI of E10 showed a significant decrease with E8 only (*p* < .001). In general, PDI values ranged from 0.4 to 0.8. No significant changes were observed in ZP (–22.07 up to –23.84) as shown in Figure 2(g). EE% showed 79.5%–83.84% as shown in Figure 2(h). Drug entrapped was maximized in E10 as a result of higher drug amount. As a higher drug amount affected PS and PDI, E10 with superior encapsulation efficiency was selected to fulfill the study objectives. However, additional optimization will be applied. Cooper and Harirforoosh upon studying the effect of formulation variables on celecoxib nanoparticles evaluated the effect of different drug amounts on entrapment capacity. Results showed that increasing drug amount triggers PL activation to enhance drug solubility and system stability, reduce drug leakage, and supported higher entrapment (Cooper & Harirforoosh, 2014). Celecoxib belongs to BCS Class II which is similar to atorvastatin as both revealed close solubility attributes. On the other hand, higher ATO-EMLs encapsulation leads to PS increment in E10 as previously reported (Vyas et al., 2006).

#### 3.2.6. PC amount

PC amount was studied to optimize E10. A gradual decrease in PS **(**Figure 2(i)**)** and PDI (Figure 2(j)) were observed with increasing PC amount. At higher PL amounts (E12), PS was 520.7 nm, which was significantly lower than E11 (837.1 nm) (*p* < .01). While PDI values for E10, E11, and E12 were 0.81, 0.98, and 0.58, respectively. E12 appeared significantly lower than E10 (*p* < .05), and E11 (*p* < .01). Similar ZP was recorded (–19.95 to –22.07 mV) as shown in Figure 2(k). The entrapped percent revealed that E11 (62.11%) was significantly lower than E10 and E12 with 83.8% and 81.5%, respectively (*p* < .05) as shown in Figure 2(l). Therefore, E12 was selected due to PS and PDI was the lowest values at highest PC amount (90 mg). As mentioned in the study by Abo El-Enin et al. upon studying an emulsome system loaded with eletriptan hydrobromide using PC in different molar ratios with compritol, a reduction in PS was reported with a higher PC amount as more emulsome vesicles may be formed (Abo El-Enin et al., 2022). These findings agreed with reported results of E12. In addition, Raza et al. explained that the emulsification properties of PL are supposed to be applied in a concentration-dependent manner (Raza et al., 2013).

#### 3.2.7. Lipid amount

**Figure 3.**
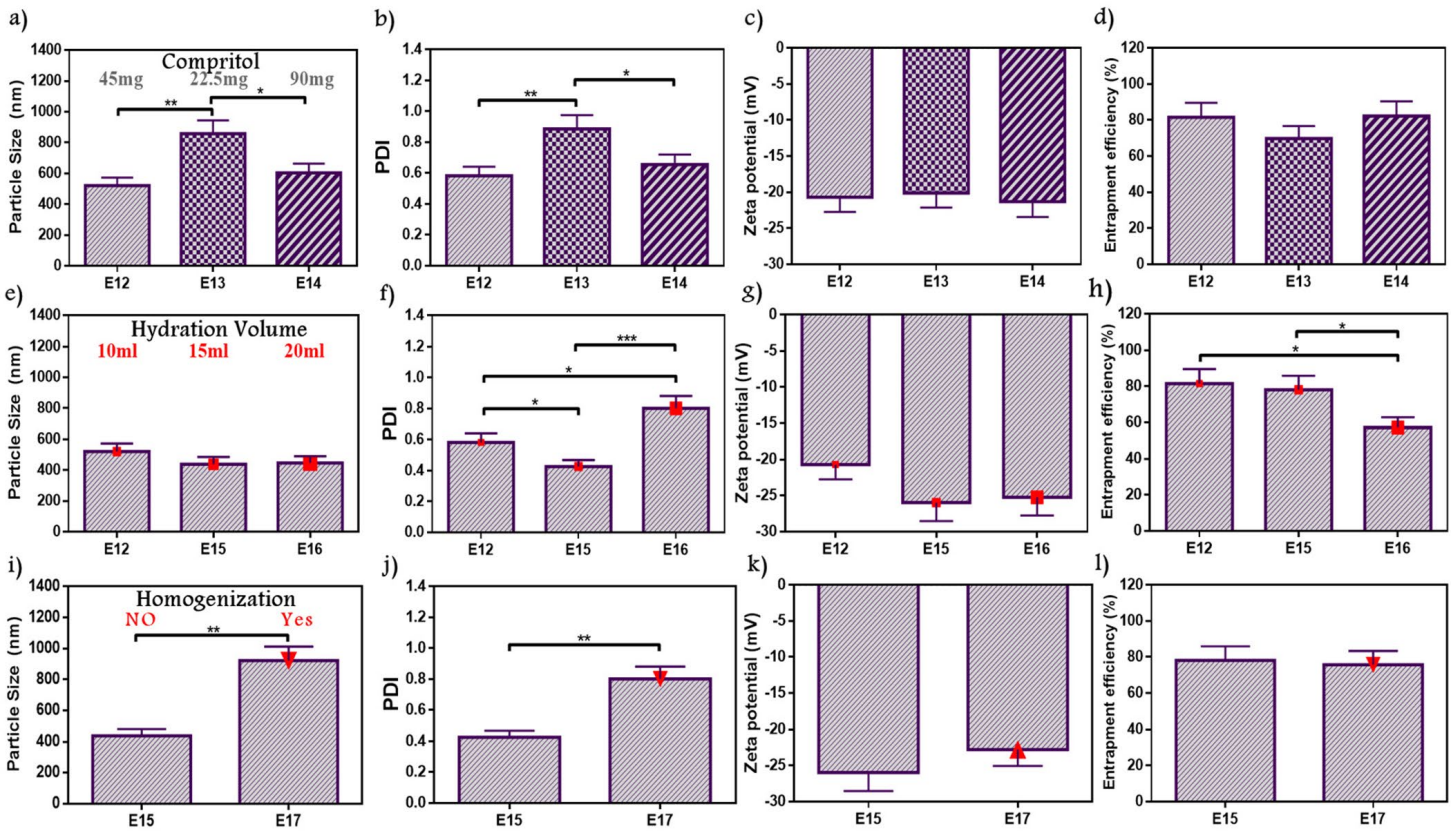
Emulsome evaluation: factor 7; effect of lipid (compritol) amount on (a) particle size, (b) polydispersity index, (c) zeta potential, and (d) entrapment efficiency percent; E12 was selected according to particle size, and polydispersity index, factor 8; the effect of hydration volume on (e) particle size, (f) polydispersity index, (g) zeta potential, and (h) entrapment efficiency percent; E15 was selected according to polydispersity index, and entrapment efficiency, factor 9; the effect of homogenization on (i) particle size, (j) polydispersity index, (k) zeta potential, and (l) entrapment efficiency percent; E15 was selected according to particle size, and polydispersity index. Data are represented as mean ± SD (*n* = 3; *p < .05, **p < 0.01, and ***p < .001).

The lipid core was inspected by adjusted Compritol weight in E13 and E14 in comparison with E12 as shown in Figure 3(a–d). PS ranged from 520.7 to 858.2 nm, whereas PDI ranged from 0.58 to 0.89. E12 acquired a significantly lower PS and PDI than E13 (*p* < .01), it was an inconsiderable difference between E12 and E14 but with preferable results to E12 as it contains the minimal lipid amount with reduced PS (520.7 nm) and PDI (0.58) values. No significant difference showed in ZP (–20.12 to –21.31) and EE% (69.73–82.21%). As a result, E12 with lipid:PL ratio (0.5:1) exhibited the most appropriate features so it was selected for further optimization conditions. Dubey and Vyas evaluated emulsome for a lipophilic drug by studying different factors. Thus upon studying several PL to lipid ratio, a variation in PS values were obtained which indicated that lipid amount and its relative ratio can affect nanosized range and stabilization (Dubey & Vyas, 2021). These findings agree with the current selection.

#### 3.2.8. Hydration volume

During vesicle preparation, the volume of deionized water used to hydrate the formed film layer can affect ATO-EMLs system attributes. As shown in Figure 3(e), PS ranged from 437.8 to 520.7 nm without significant difference in the studied formulations (E12, E15, and E16) with elevated hydration volumes of 10 mL, 15 mL, and 20 mL, respectively. E15 revealed minimal PDI (0.43), and it showed significant differences with E12 as it was 0.58 (*p* < .05), and E16 was 0.8 (*p* < .001) as shown in Figure 3(f). ZP ranged from –20.68 to –25.95 with no significant difference over the formulations. Furthermore, E15 showed 78.15% of the drug entrapped which is significantly higher than E16 with only 57.2% entrapment (*p* < .05) (Figure 3(g,h)). E15 with a volume of 15 mL was selected due to lower PDI. Danaei et al. studied the lipidic nanocarriers, where vesicle PS homogeneity and stability influenced drug distribution in target tissues during the application. Accordingly, lower PDI indicates higher distribution. Moreover, it was noticed that nanocarrier composition and solvent properties affect PDI (Danaei et al., 2018). This explanation agreed with current findings.

#### 3.2.9. Homogenization duration

The effect of homogenization was studied in E17 and compared with E15 (without homogenization) as shown in Figure 3(i,j). Using homogenizer showed a significant increase in PS from 437.8 to 920.5 nm and PDI from 0.43 to 0.8 after homogenization (*p* < .01). However, ZP and EE% showed no significant differences (Figure 3(k,l)). ZP was –25.95 and –22.78 mV and EE% was 78.15% and 75.79% for E15 and E17, respectively. As a result, emulsome formula without homogenization (E15) was more suitable. Putri et al. studied the effect of homogenization cycles and pressure during the evaluation of nanoparticle formulation for an antitumor agent. This study determined that homogenization properties may influence PS and PDI. Increasing PS may occur due to the cohesion of very small parts passing through the homogenizer valve at elevated pressures (Putri et al., 2021).

#### 3.2.10. Probe sonication duration

**Figure 4.**
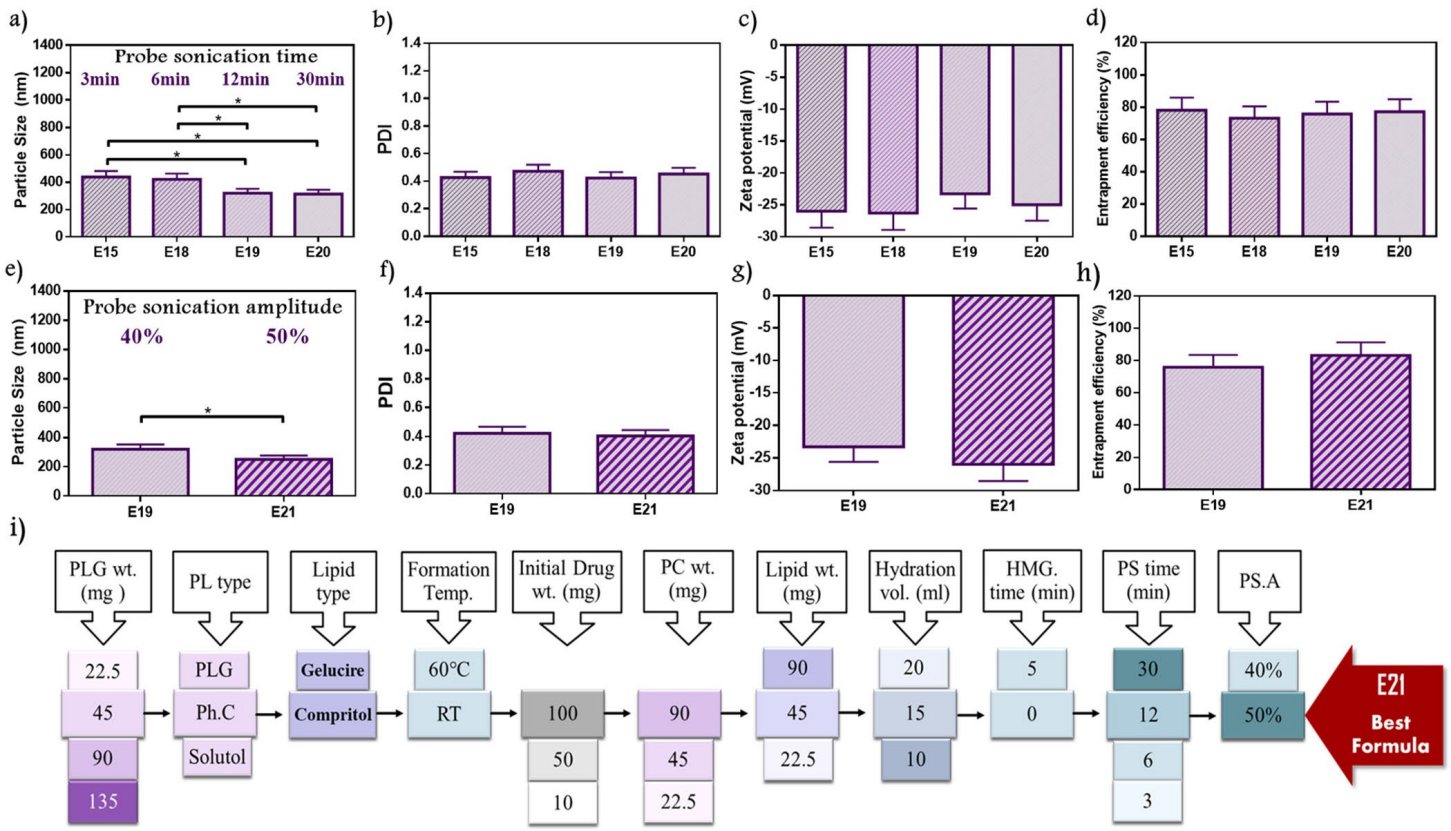
Emulsome evaluation: factor 10; effect of probe sonication time on (a) particle size, (b) polydispersity index, (c) zeta potential, and (d) entrapment efficiency percent; E19 was selected according to particle size, factor 11; the effect of probe sonication amplitude on (e) particle size, (f) polydispersity index, (g) zeta potential, and (h) entrapment efficiency percent; E21 was selected according to particle size, and elected as the best formula for further characterization, (i) Diagrammatic illustration of OFAT method represented the 11 factors with variables and chosen elements. Data are represented as mean ± SD (*n* = 3; *p < .05).

The duration of probe sonication was studied as shown in in Figure 4(a–d). Data showed a decrease in PS with the long duration. E19 (319.5 nm) was significantly lower than E15 (437.8 nm) and E18 (420.5 nm) (*p* < .05), while no significant difference from E20 (313.1 nm). Moreover, PDI was in the range of 0.42–0.47 without significant changes. All formulae studied under this factor revealed ZP ranging from –23.23 to –26.27, and EE% from 73.23 to 78.15%. The sonication for 12 min (E19) and 30 min (E20) demonstrated similar results. Therefore, E19 with shorter duration was selected. As reported by Fahmy et al. during the study of the encapsulation of febuxostat into an emulsome system, ultrasonication time was incorporated as a variable in an experimental design for targeting size minimization, results concluded that ultrasound mechanical waves of probe sonication applied high energy on vesicle system resulted in time-dependent size reduction (Fahmy et al., 2020).

#### 3.2.11. Probe sonication amplitude

All previously studied formulae were exposed to 40% probe amplitude. In the current factor, E21 with a higher amplitude value reaching 50% was investigated and compared to E19 (Figure 4(e–h)). E21 showed a significant decrease in PS as it was 250.5 nm (*p* < .05). On the other hand, no significant difference was observed in PDI of E19 and E21 with values of 0.42 and 0.4, respectively. Recorded ZP for E19 and E21 showed nearby values that were about –23.23 mV, and –25.93 mV, respectively. Furthermore, the EE% measured for E19 was 75.82% while 83.12% for E21. Therefore, E21 was selected because of its lower PS. Silva et al. studied ultrasound parameters’ influences on vesicle preparation representative of the power input (amplitude) and distance from the tip. Upon evaluating three power inputs (23%, 30%, and 40%), reductions in PS and PDI were observed by dynamic light scattering (DLS), that’s explained as the enforcement of higher power to the system applied greater shear forces causing a decrease in the measuring parameters (Silva et al., 2010).

In conclusion, 11 factors were investigated using one factor at a time design as represented in Figure 4(i). From the 11 factors, three factors (lipid types, lipid amount, and homogenization) did not improve formulation responses, while the other factors significantly improved formulation responses. E21 was selected as the best formula that achieved the predetermined specifications.

### 3.3. Characterization of optimum formula

According to the revealed data from the OFAT, E21 was selected as the best and subjected for further characterization.

#### 3.3.1. Vesicles morphology

**Figure 5.**
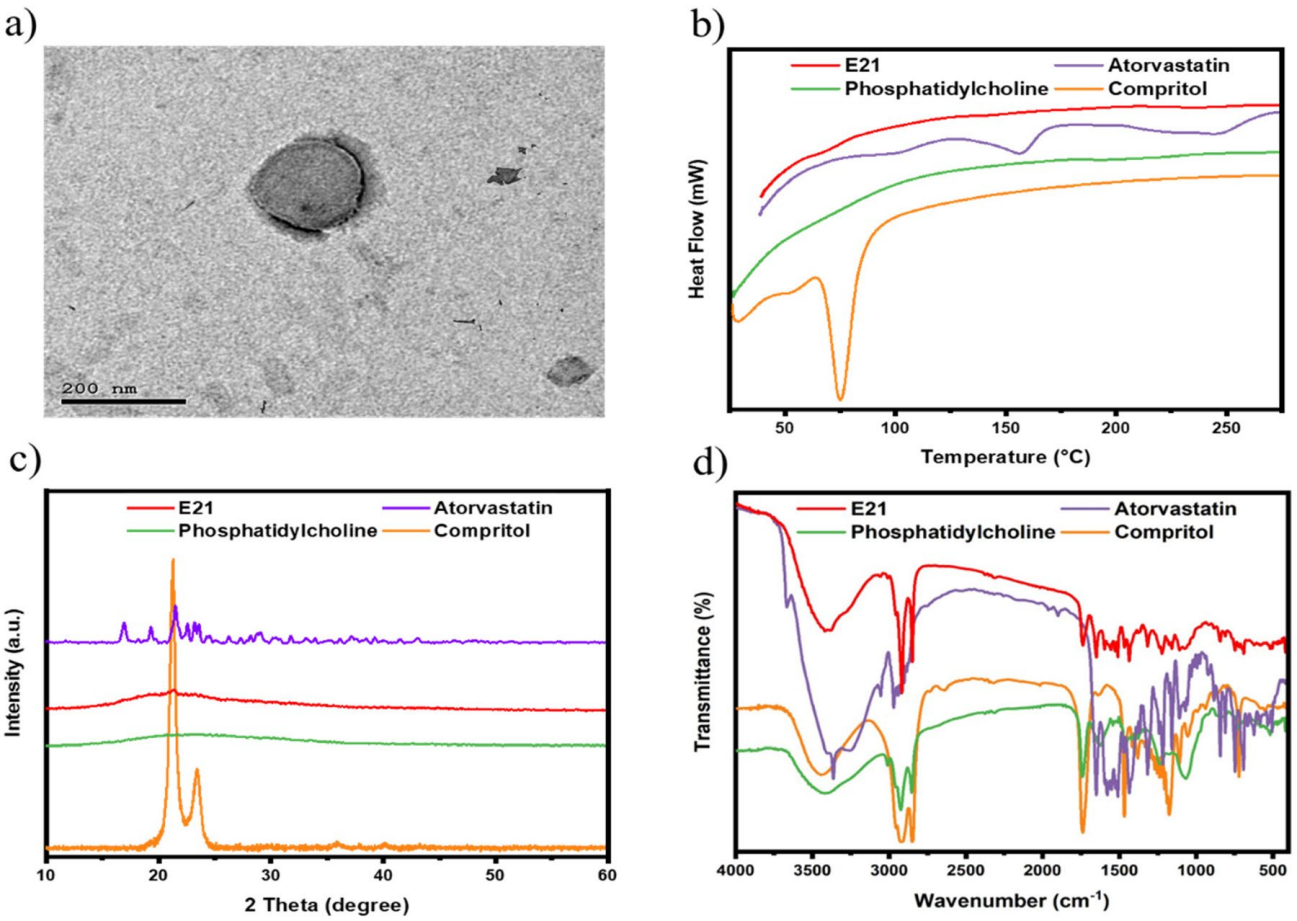
Characterization of the optimum formula: (a) TEM image of emulsome vesicle system showed spherical uniform shape with size around 200 nm, physicochemical characterization applied on free atorvastatin, compritol, phosphatidylcholine, and optimum emulsome formula (E21): (b) Differential scanning calorimetry thermograms, (c) X-ray diffraction analysis thermograms, and (d) Fourier transforms infrared spectra; prove vesicle formation and drug entrapment.

The microscopic examination of the optimized formulation using TEM in Figure 5(a) showed a bilayer spherical shape with a smooth uniform surface as a result of PC outer shell with inner Compritol core. The size was seen to be around 200 nm. Paliwal et al. reported emulsome as a carrier for methotrexate, the TEM photograph showed slight light rounded lecithin shell layer with a darkened inner lipid core (Paliwal et al., 2009), which appeared similar to the presented results.

#### 3.3.2. Differential scanning calorimetry

DSC was carried out to confirm the successful formation of ATO-loaded EMLs. As shown in Figure 5(b), DSC thermograms of atorvastatin showed two endothermic peaks at about 156 °C and 243.7 °C, which is in agreement with previous report (da Silva et al., 2016). The crystalline atorvastatin acquired two peaks corresponding to ATO melting and decomposition points. Moreover, Compritol appears with a sharp endothermic peak at approximately 75 °C. ATO characteristic peaks disappeared in the E21 thermogram, which proved diffusion of ATO in the core–shell emulsome system.

#### 3.3.3. X-ray diffraction analysis

As shown in XRD patterns (Figure 5(c)), a natural crystalline form is remarked by characteristic high-intensity diffraction peaks for ATO-free drug and Compritol. As reported by Abootorabi et al., ATO revealed several diagnostic peaks at 2*θ*: 16.93°, 19.33°, 21.55°, 22.59°, 23.21°, and 23.59° (Abootorabi et al., 2022). Moreover, Compritol showed two sharp characteristic peaks at 21.29° and 23.49°, which is due to lipidic polymorphism (Jagdale et al., 2011). Otherwise, ATO and compritol peaks disappeared in the E21 thermogram as a result of the transformation of their physical state from crystalline to amorphous form when incorporated into the vesicle system.

#### 3.3.4. Fourier-transforms infrared spectroscopy

The formation of E21 was chemically inspected by investigating the characteristic peaks of used ingredients in FTIR spectra. FTIR spectra of Atorvastatin, Compritol, PC, and optimized ATO-EMLs formulation are shown in Figure 5(d). The FTIR spectrum of pure ATO showed many distinctive peaks mainly at about 3666.7 cm^−1^ (non-hydrogen-bonded O-H), 3363.9 cm^−1^ (N-H stretching), 3257.8 cm^−1^ (O-H stretching), 2970.4 cm^−1^ (C-H stretching), 2920.2 cm^−1^ (C-H, aromatic), 1651.1 cm^−1^ (asymmetric C = O stretching), 1577.8 cm^−1^ (N-H bending), 1508.3 cm^−1^ (C-N stretching), 1435 cm^−1^ (O-H bending), and 1381 cm^−1^ (C-O stretching) of the carboxyl group as reported by Ghanem et al. (2021). Compritol acquired a broad band between 3650 cm^−1^ and 3100 cm^−1^ due to (OH stretching). The appearance of most characteristic peaks of the ATO in E21 spectra may indicate that the drug was entrapped in the lipid mixture.

### 3.4. Stability study

**Figure 6:**
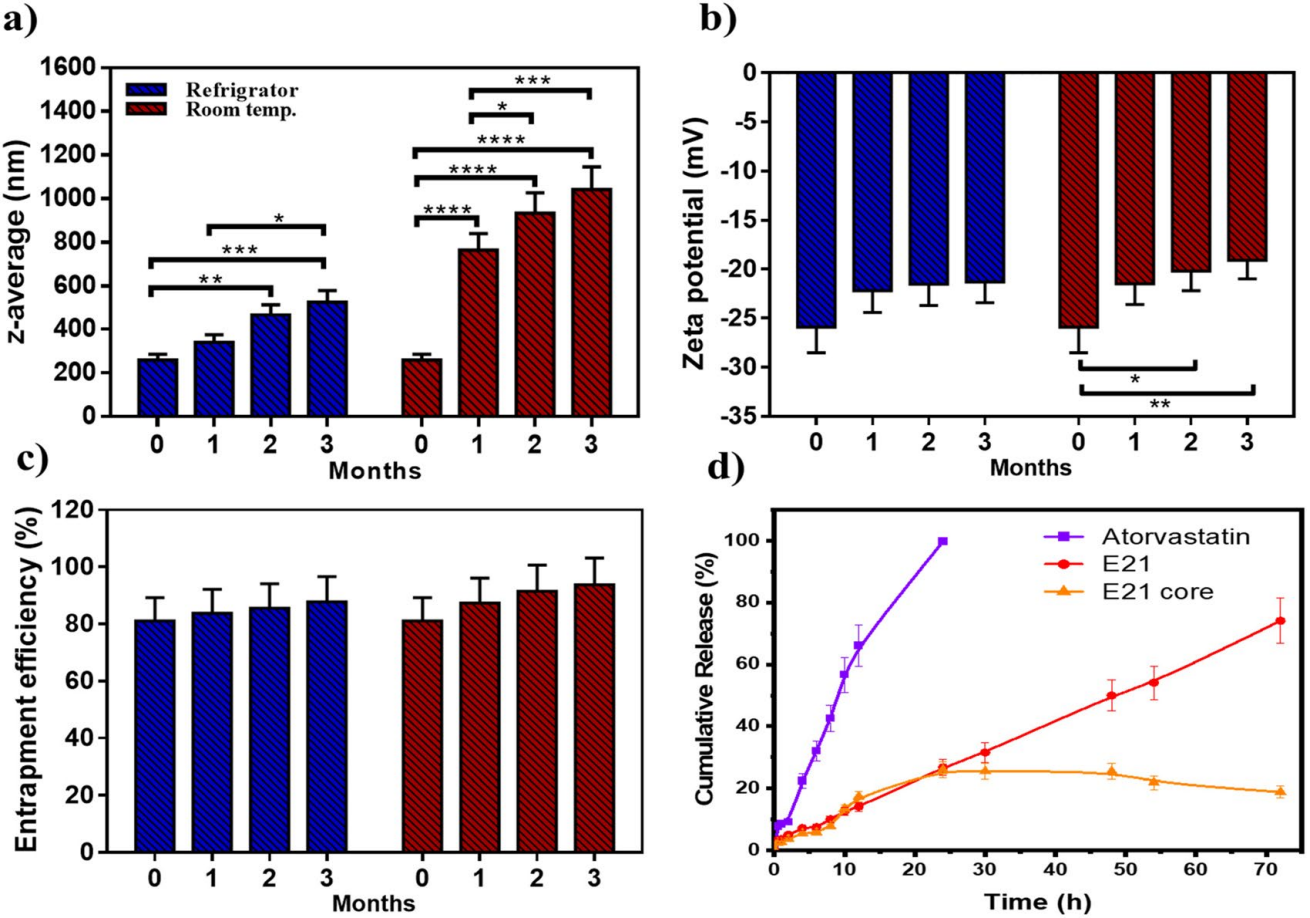
**.** Stability study for 3 months, evaluated refrigerating (4 °C) in comparison to room temperature which represented as (a) particle size, (b) zeta potential, and (c) entrapment efficiency percent; storage at refrigerator was preferred as emulsome system reflected higher stability attributes, (d) Release study illustrated as cumulative percentage of atorvastatin free drug in comparative to loaded emulsome (E21), as the later showed controlled drug release, in addition, E21 core release profile was studied to evaluate the amount of drug entrapped in the core and the shell separately; diagram showed that about 25% of the total entrapped drug (80%) was revealed to core entrapment. Data are represented as mean ± SD (*n* = 3; *p < .05, **p < .01, ***p < .001, and ****p < .0001).

The physical stability of emulsomes was conducted at 4 ± 1 °C and 25 ± 1 °C, where PS, ZP, PDI, and EE% of ATO-EMLs were tested. Measurements were taken immediately after prepared, and then monthly for 3 months. At 4 °C, the mean PS (Figure 6(a)) appeared with slightly elevated values ranging from 259.5 to 526.3 nm (Figure 6(a)), the fresh sample showed a significant increase in PS after two (*p* < .01), and three (*p* < .001) months. Size enlargement could be explained by the accumulation and agglomeration of the small vesicle system (Elakkad et al., 2021). Size distributions appeared with no significant change as PDI ranged from 0.40 to 0.56. Moreover, ZP and EE% showed no significant variation as shown in Figure 6(b,c) with no noticeable change in physical appearance. In contrast, at 25 °C significant variations were recorded in PS ranging from 259.5 to 1042.2 nm (Figure 6(a)) and PDI at the range of 0.4–0.95. In addition, ZP revealed a significant decrease when comparing the fresh sample with stored one after two months (*p* < .05), and three months (*p* < .01) which may affect system stability. In conclusion, emulsome should be stored in a refrigerator as storage temperature with minimal effect on physical properties as previously reported (Ucisik et al., 2015). A stabilizer may be favorable for the formulation to increase emulsomes stability which will be considered in future work.

### 3.5. *In vitro* release study

An *in vitro* release study was performed for free ATO, ATO-EMLs system, and ATO-EMLs previously treated with ethanol to measure the release profile. The dissolution media was selected to maintain the sink condition as previously reported by Mahmoud et al., where propylene glycol as co-solvent was used for ATO-loaded transfersomes (Mahmoud et al., 2017). As shown in Figure 6(d), free ATO showed rapid solubilization over the first 12 h about 66% was dissolved followed by a complete dissolution achieved after 24 h. On the other hand, the ATO-EMLs system revealed a slow release indicating a controlled and gradual release profile that exhibited almost 75% released after 72 h. Furthermore, the shell of E21 was removed by ethanol and the release profile was measured. Only 25% of the drug was released from the core of the emulsome system within 72 h. These findings revealed that most of the drug was entrapped in the shell part. The release profile of drug suspension and ATO-EMLs was following a zero-order kinetics as it considers the best fitting model due to higher *R*^2^ that were 0.9910 and 0.999, respectively. Zero order describes the system when the drug appeared to release slowly (Elbaz et al., 2016), and the release rate is concentration independent.

### 3.6. *In vivo* permeation visualization using confocal laser scanning microscopy

**Figure 7.**
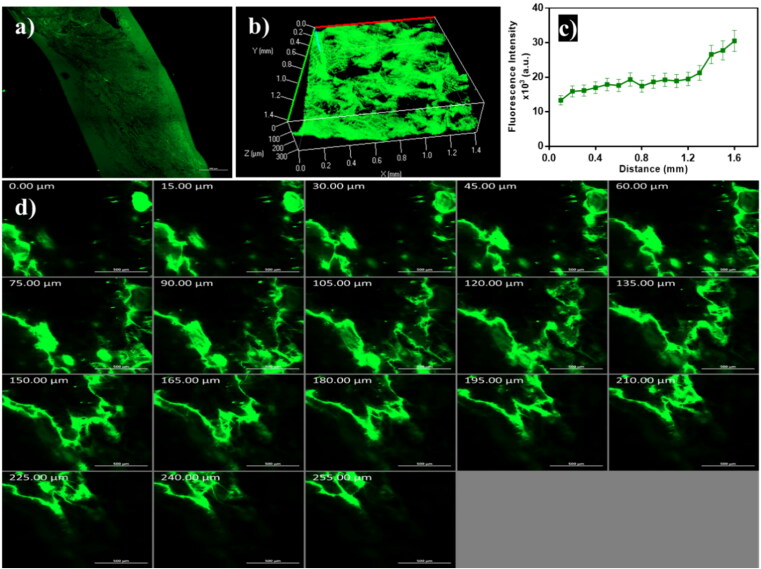
Confocal laser scanning microscopy images of emulsome (a) tile x-y image of skin treated with FDA-loaded emulsome, (b) 3D plot of the z-stack images, (c) quantitative measurement of fluorescence intensity profile across skin layers, and (d) Z-stack images of the skin section from 0 µm to 255 µm with 15 µm increments; showed that penetration occurred across different skin layers as remarked with high fluorescence intensity.

As demonstrated in Figure 7(a), the FDA-loaded emulsome penetrated across different skin layers as remarked with high fluorescence intensity. The 3D image compiled by Z-stack of *x*-*y* images, which showed a homogeneous distribution of emulsomes in skin layers (Figure 7(b)). The intensity of the fluorescent dye was measured, and data revealed that the dye reached deep skin layers (Figure 7(c)). The overall skin permeation can take place through the transcellular, paracellular, and trans-appendageal routes (Ghasemiyeh & Mohammadi-Samani, 2020). Favorable oily components in the sebaceous gland attract lipid base formulation to penetrate the skin through the follicular route. Fluorescence accumulation in the epidermis and dermis layer was monitored to simulate the pathway of ATO-loaded EMLs revealed to skin permeation (Figure 7(d)). Noticed that vesicles with a nanosize range enhanced deep penetration and promoted antifungal drug therapy (Hussain et al., 2016).

### 3.7. *In vitro* microbiology study

The susceptibility of isolated *C. albicans* strains to ATO-loaded EMLs, and blank EMLs was evaluated through the determination of MIC and zone of inhibition, in comparison to a standard commercial drug (Terbinafine HCl).

#### 3.7.1. Micro-Well dilution method

**Figure 8.**
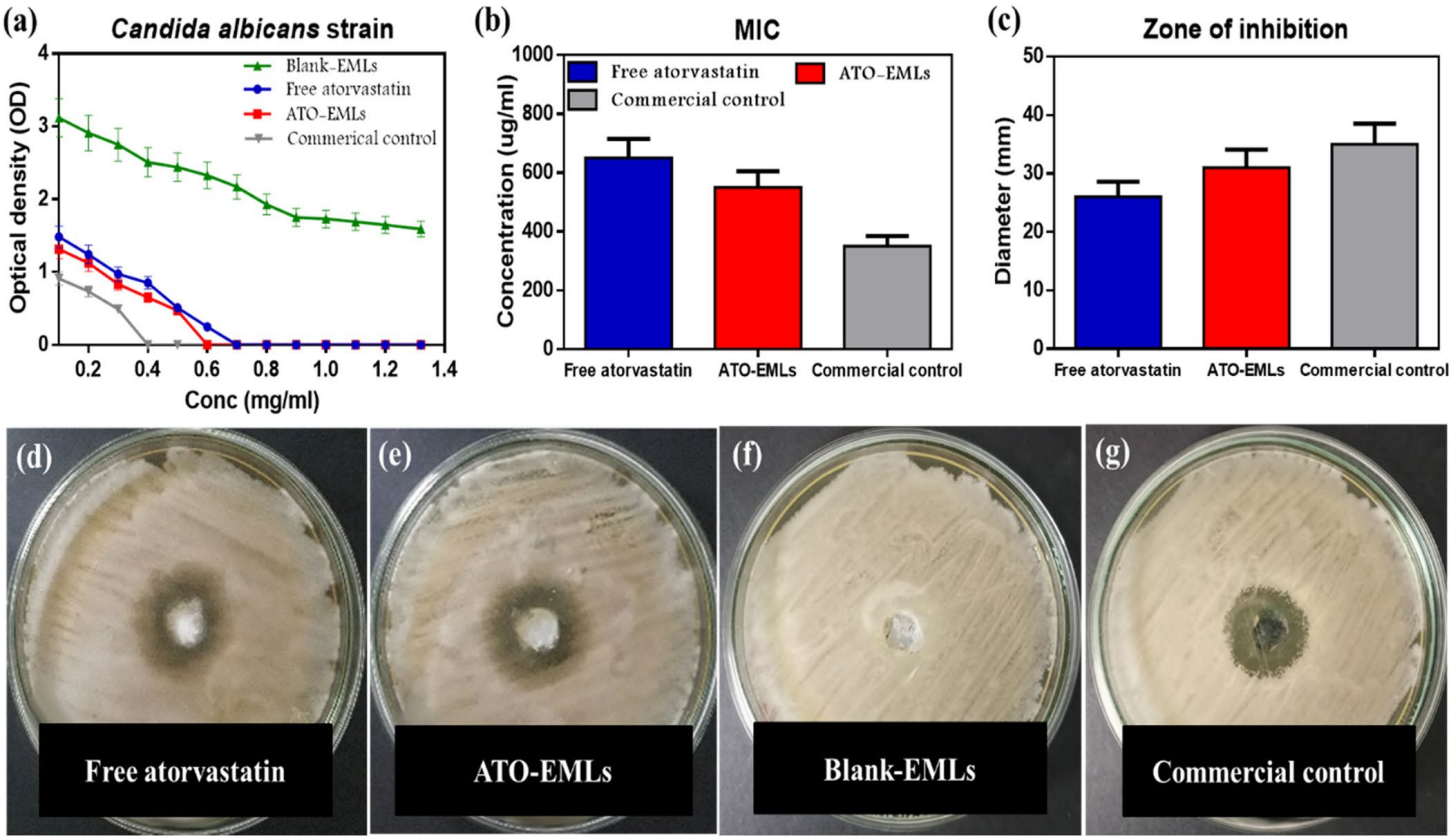
*In-vitro* microbiology studies: (a) optical density of bacterial growth illustrated against the diluted concentration of investigated samples to determine MIC; blank-EMLs revealed with no antifungal activity, the susceptibility of isolated *C. albicans* strains to atorvastatin-free dug suspension, ATO-loaded EMLs in addition to commercial control was evaluated through measured (b) MIC values, (c) diameters of inhibition zone, inhibition zones are shown in agar plate for (d) free atorvastatin, (e) ATO-EMLs, (f) blank-EML; showed no zone of inhibition, (g) commercial control.

The optical density determined for the various dilutions of the formulae incubated with *C. albicans* is represented in Figure 8(a,b). MIC values were measured for each sample. MIC of ATO suspension was 650 µg/mL, while incorporated drug in the emulsome system reduced the value to 550 µg/mL, otherwise, blank-EMLs observed no antifungal activity. The MIC of commercial drug was 350 µg/mL.

#### 3.7.2. Well diffusion method

The diameter of the inhibition zone was measured at MIC in Figure 8(c–g). ATO-EMLs were more sensitive than free drug as the diameters increased from 26 mm to 31 mm, while blank-EMLs showed no zone of inhibition. The standard commercial drug showed an inhibition zone of 35 mm. Mahmoud et al. studied the *in vitro* anticandidal activity of atorvastatin alone and in combination with other conventional antifungal drugs, the microdilution method and diffusion method both proved the antifungal activity of atorvastatin (Mahmoud et al., 2021)

### 3.8. *In vivo* study

#### 3.8.1. Skin irritation test

**Figure 9.**
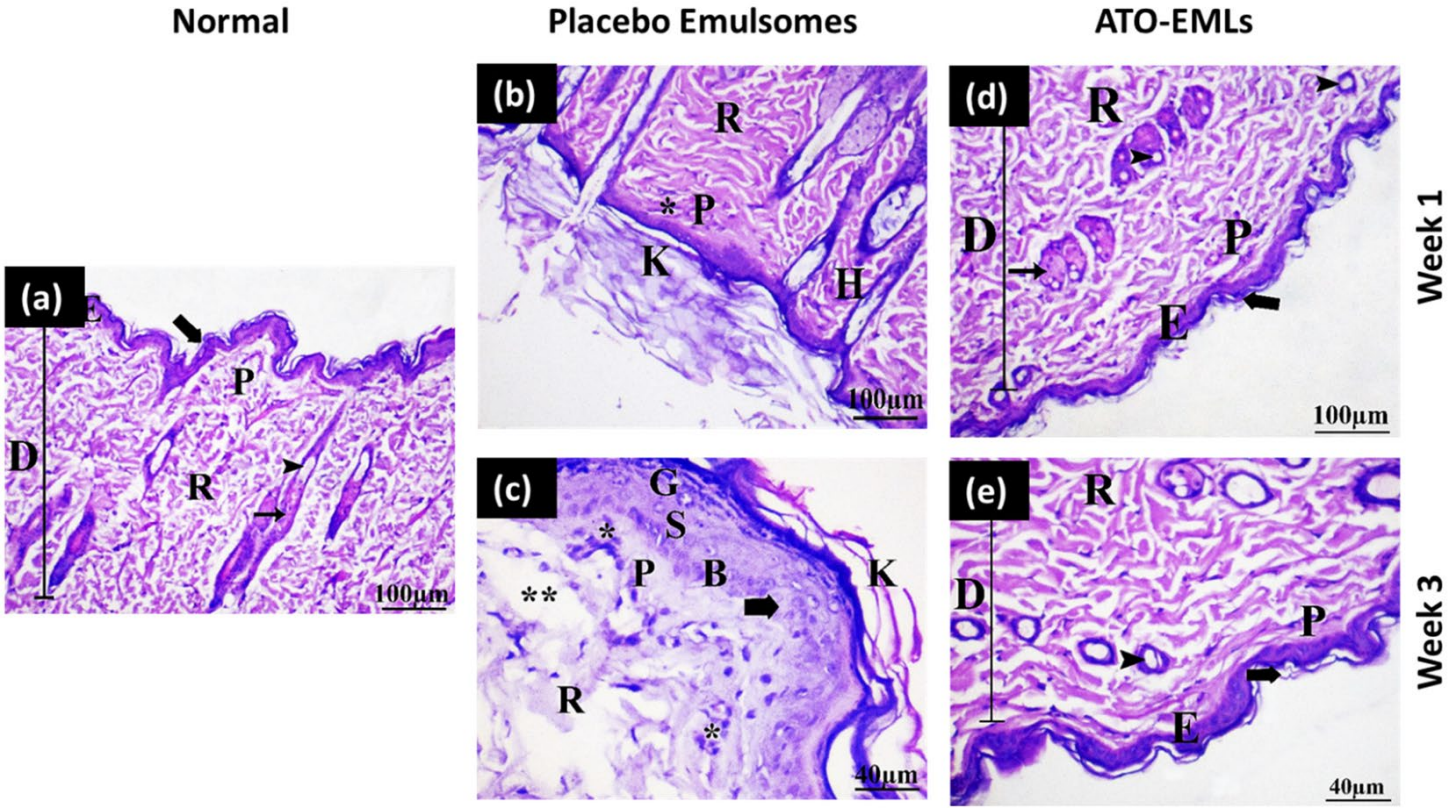
*In-vivo* skin irritation test: (a) Normal skin: This shows the two layers of the skin, the epidermis (E) and the dermis (D). The dermis shows an outer papillary layer (P), an inner reticular layer (R), and hair follicles (arrowhead) associated with sebaceous gland (arrow). The stratum corneum consists of many layers of flattened non-cellular acidophilic keratin scales (bold arrow). (b) Placebo emulsomes after 1 week: Hyperkeratosis (K), increased thickness of the stratum corneum with non-nucleated corneocytes, due to alteration in epidermal cell turnover and differentiation of superficial keratinocytes. Hyperkeratosis is a common sequel of chronic epidermal disease and is caused by increased turnover of epidermal cells or decreased desquamation of corneocytes. Hyperkeratosis can be a sign of skin irritancy. Perifollicular and intrafollicular infiltration of neutrophils, lymphocytes, and mononuclear cellular infiltration in the dermis (*). (c) Placebo emulsomes after 3 weeks: The epidermis (E) and dermis (D) are seen in this figure. Hair follicles (arrowhead) related with sebaceous glands (arrow) can be seen in the dermis’ outer papillary layer (P) and an inner reticular layer (R). Flattened non-cellular acidophilic keratin scales cover most of the stratum corneum (bold arrow). **(**d**)** ATO-EMLs after 1 week: The dermis shows focal areas of collagen loss or empty space (**) of variable sizes. Increased cellular proliferation of the epidermis and epithelial hyperplasia is the most common spontaneous, non-neoplastic lesion of the skin (bold arrow). Hyperkeratosis (K). Perifollicular and intrafollicular infiltration of neutrophils, lymphocytes, and mononuclear cellular infiltration in the dermis (*). (e) ATO-EMLs after 3 week. These are the two layers that make up the skin. The epidermis (E) and the dermis (D) may be seen here. The dermis can be broken down into two distinct layers: the outside papillary layer (P), the inner reticular layer (R). There was hair follicle (arrowhead). The stratum corneum is made up of multiple layers of keratin scales that are non-cellular and acidophilic and have been flattened (bold arrow). Mononuclear cellular infiltration in the dermis (*) was detected.

Histopathological examination of skin specimens from different groups was conducted to test the side effects of the developed formula. For comparison, a normal skin specimen was obtained from a normal rat. In Figure 9(a), the epidermis and dermis of the control group were separated by an irregular basement membrane in thin skin sections. The epidermis was found to be made up of four layers of stratified squamous keratinized epithelium. The stratum basale cells were a single layer of cuboidal to low columnar cells that rested on the basement membrane. The stratum spinosum was made up of many layers of polyhedral keratinocytes. Keratinocytes in the stratum granuolosum appeared flattened, with many strongly basophilic keratohyalin granules. A cornified layer of flat, homogeneous keratinized non-nucleated cells created the stratum corneum. The papillary layer of the dermis was displayed under the epidermis as a thin layer of loose connective tissue that formed the substance of the dermal papillae. The reticular dermis lay deep to the papillary dermis, not clearly divided from it, and seemed thicker and less cellular than the papillary dermis. The dermis was densely packed with hair follicles and sebaceous glands.

The placebo formula group was examined after 1 and 3 weeks. Figure 9(b) shows the histological structure of skin treated with a placebo formula after 1 week. Some places were shown to be covered by a thin epidermis consisting of 1–3 cell layers in thickness. The epidermis was thick, and the keratin layer was thickened by a mild degree. The underlying dermis with mild inflammatory cell invasion was observed. The dermis layer revealed normal organization without fiber accumulation. No hair follicles and sebaceous glands hyperplasia were evident. After 3 weeks, the skin demonstrated some regions with a thin epidermis. The keratin layer has been greatly (moderate degree) increased in thickness. There was mild hyperplasia of epidermal cells. The dermis was infiltrated by a moderate degree of inflammatory cells. Some hair follicle cells with follicular bulges were found. The dermis had a mild rate of empty space as shown in Figure 9(c). So, after three weeks period from the application of the placebo formula, the skin revealed an increase in hyperkeratosis degree and inflammatory cells infiltration and there was a slight improvement in epidermal hypertrophy compared with one week period. This indicates that the formula might have a slight undesirable effect on skin layers. This could be returned to the presence of surfactant (PL) in the formula.

The effect of drug-loaded formula (ATO-EMLs) on the skin was also investigated after 1 and 3 weeks. Figure 9(d) shows the effect of ATO-EMLs after 1 week. The dermis comprised large bundles of collagen fibers (moderate degree) that were seen in the papillary layer of the dermis. The keratin layer was moderately thick. Along with the hypertrophy of the epidermal cells, there was an apparent increase in the number of epidermal cells (hyperplasia). The dermis beneath the skin exhibited disordered fibers as well as a mild level of inflammatory cell infiltration. In some regions, hyperplasia and proliferation of some hair follicle cells were seen. In the dermis, there was an empty spot. A moderate degree of sebaceous gland hyperplasia was observed. After 3 weeks, a thin epidermis of 1–3 cell layers thick covered some regions (moderate degree). A mild thick keratin layer was observed. In the dermis, with mild inflammatory cells, the dermis had many hair follicles without sebaceous glands. In some regions, mild follicular bulge and mild sebaceous gland hyperplasia were observed. No fibrosis and empty spaces were found in the dermis layer as shown in Figure 9(e). The histopathological findings were also evaluated using a semiquantitative method by scoring five fields for each time point. Each field was subjected to evaluation against 10 parameters, where three grades were used for evaluation (no abnormality, slight, and mild). The average of each grade was calculated for each time point. As shown in Table 2, the placebo group after 1 week showed that most parameters were normal with an average of 3.5. After 3 weeks, the skin showed normal to slight changes with a total average of 3.5. ATO-EMLs group showed no abnormality with an average of 3.4 and 3.5 for 1 week and 3 weeks, respectively. These findings confirm the safety of the developed formula. In conclusion, following three weeks interval from the usage of ATO-EMLs, the skin showed improvement in the grade of hyperkeratosis, inflammatory cell infiltration, epidermal hypertrophy, fibrosis, and follicle hyperplasia compared with a one-week interval. This implies that the formula might have not an unfavorable effect on skin layers and the skin undergoes recovery after a period of treatment stops. This effect could be a result of the presence of ATO with an anti-inflammatory effect.

**Table 2.** Scoring of histopathological findings of *in-vivo* irritation test for placebo and ATL-EMLs groups after 1 and 3 weeks.

Groups	Placebo formula						ATO-EMLs					
Time	Week 1			Week 3			Week 1			Week 3		
Grade	–	+	++	–	+	++	–	+	++	–	+	++
1. Hyperkeratosis	2	3	0	0	2	3	1	2	2	2	2	1
2. Detached & fragmented SC	3	2	0	2	3	0	3	2	0	3	2	0
3.Inflammatory cells infiltration	1	4	0	2	3	0	1	2	2	2	2	1
4. Thin epidermis	2	3	0	5	0	0	3	2	0	2	1	2
5. Thick epidermis (hypertrophy)	2	3	0	0	1	4	3	2	0	5	0	0
6. Empty space / dermis	5	0	0	2	3	0	3	2	0	5	0	0
7. Hyperplasia of the hair follicle	5	0	0	2	3	0	5	0	0	3	2	0
8. Destruction of the fatty layer	5	0	0	2	3	0	5	0	0	5	0	0
9. Fibrosis/dermis	5	0	0	1	0	4	5	0	0	5	0	0
10. Sebaceous gland hyperplasia	5	0	0	1	0	4	5	0	0	3	2	0
Average	3.5	1.5	0	1.7	1.8	1.5	3.4	1.2	0.4	3.5	1.1	0.4

The number of examined fields evaluated for each time point was 5. Grade of histopathological findings: – (no abnormality), + (slight), ++ (mild).

#### 3.8.2. Microbiological study

**Figure 10.**
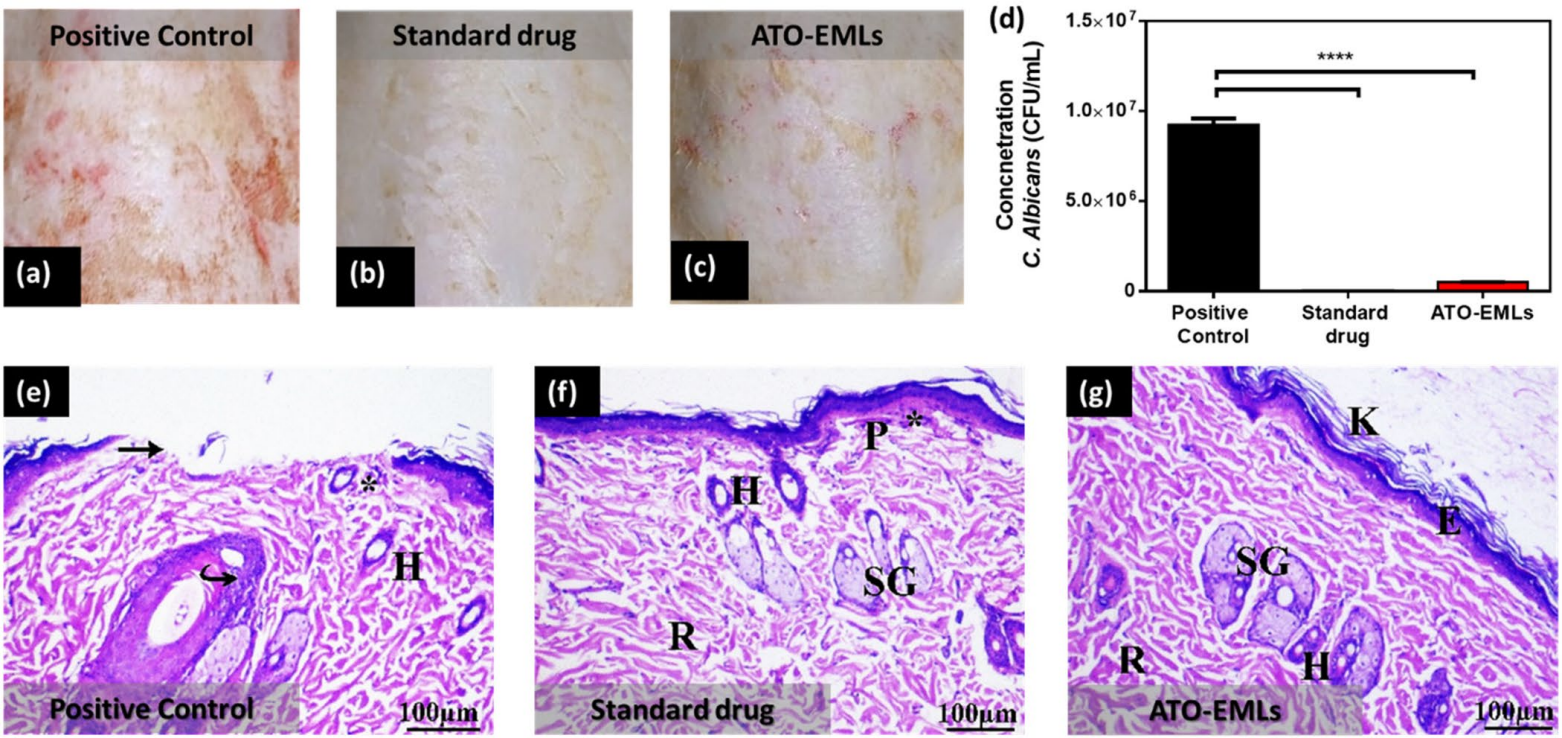
*In vivo* microbiological study: photographic images of the infected skin area of rat dorsal (a) positive control group, (b) standard drug group, and (c) emulsomes ATO-EMLs group. (d) *C. albicans* concentration in CFU/mL of different groups (****p < .0001). Histopathological evaluation: (e) Positive group: Perifollicular and intrafollicular infiltration of neutrophils, lymphocytes, and mononuclear cellular infiltration in the dermis (*). Hair follicles (H) with an absence of associated sebaceous glands are observed. Focal areas of epidermal discontinuation (arrow) due to loss of superficial epidermal cell layers (erosion). Increased cellular proliferation (hyperplasia) of some hair follicles as a follicular bulge (curved arrow). (f) Standard drug group: perifollicular and intrafollicular infiltration of neutrophils, lymphocytes, and mononuclear cellular infiltration in the dermis (*). (g) ATO-EMLs group: hyperkeratosis (K) increased the thickness of the stratum corneum with non-nucleated corneocytes, due to alteration in epidermal cell turnover and differentiation of superficial keratinocytes. Normal epidermis thickness (E) was observed. The dermis shows an inner reticular layer (R), hair follicles (H), and Sebaceous glands (SG).

Skin fungal infections are common in immunocompromised patients, where *C. albicans* is the common source of infection. Traditional drugs could have serious side effects. Therefore, discovering new drugs with less serious side effects had pursued by different scientists. The current study compared the effect of a commercial product as a standard drug (Terbinafine HCl) with ATO in a nanoformulation (ATO-EMLs). After 3 weeks of treatment, the skin hair was shaved with a clipper. The clinical evaluation of the infected area showed redness in the positive control group (Figure 10(a)). The standard drug group showed no signs of skin abnormality (Figure 10(b)). The ATO-EMLs group showed slight redness (Figure 10(c)). A microbiological evaluation of the infected area of each group showed that the positive control group had a viable *C. albicans* concentration of 9.23 *E* + 06 CFU/mL, which was significantly different from the standard drug group with a concentration of 4.07 *E* + 04 CFU/mL (****p < .0001) and ATO-EMLs group with a concentration of 5.00 *E* + 05 CFU/mL (****p < .0001) as shown in Figure 10(d). The standard drug group showed no significant difference from ATO-EMLs group. The histopathological evaluation was conducted to confirm the microbiological activity of the developed formula and compare the findings with the standard drug. Positive control group showed moderate hypertrophy of the epidermal cells, where there was an apparent increase in the number of epidermal cells (hyperplasia) and covered with thick stratum corneum (hyperkeratosis) by a moderate degree. Certain epidermis regions appeared with erosion changes. The underlying dermis showed mild inflammatory cell invasion. The dermis layer revealed a mild degree of atrophy in hair follicles, cyst follicles, and most follicles not associated with the sebaceous gland as shown in Figure 10(e). The standard drug group showed that the keratin layer has been greatly (moderate degree) increased in thickness. There was slight hyperplasia of epidermal cells and some region showed normal epidermal thickness. No epidermal erosion was recovered. The dermis is infiltrated by a mild degree of inflammatory cells. Some areas revealed multiple numbers of a hair follicles, and moderate levels of cyst follicles as shown in Figure 10(f). Finally, the developed formula ATO-EMLs group showed that the keratin layer was slightly thick. A specific region of the epidermis was thick. No epidermal erosion was noticed. The dermis beneath the skin exhibited a slight level of inflammatory cell infiltration. Most hair follicles were normal and associated with the sebaceous gland were seen. No cyst follicles were seen in the dermis layer. The histopathological findings were also evaluated using a semiquantitative method by scoring 10 fields for each group. Each field was subjected to evaluation against eight parameters, where four grades were used for evaluation (no abnormality, slight, mild, moderate). The average of each grade was calculated for each group. As shown in Table 3, the positive control group showed that most parameters were abnormal signs with a total average of 6 (mild–moderate). For the standard drug group, the skin showed normal to slight changes with a total average of 6.5. ATO-EMLs group showed normal to slight changes with a total average of 10. These findings confirm the efficacy of the developed formula. In conclusion, the standard drug and ATO-EMLs groups showed improvement in the grade of hyperkeratosis, inflammatory cell infiltration, epidermal hypertrophy, and the number of hair follicles in comparison to the positive control group. ATO-EMLs formula showed a good antimicrobial activity similar to the standard drug. These findings indicate that the developed formula has a desirable effect on dermal fungal infection after treatment.

**Table 3.** Scoring of histopathological findings of *in-vivo* microbiological study for positive control, standard drug and ATL-EMLs groups after 3 weeks of treatment.

Groups	Positive control				Standard drug				ATO-EMLs			
Grade	–	+	++	+++	–	+	++	+++	–	+	++	+++
1. Hyperkeratosis	0	1	4	5	2	1	4	3	7	3	0	0
2. Inflammatory cells infiltration	1	3	6	0	2	3	5	0	5	5	0	0
3. Thick epidermis (hypertrophy)	2	3	5	0	5	5	0	0	8	2	0	0
4. Atrophy	0	2	8	0	6	4	0	0	10	0	0	0
5. Number of hair follicle	10	0	0	0	1	3	3	3	7	3	0	0
6. No sebaceous gland	0	0	5	5	7	1	2	0	8	2	0	0
7. Erosion	5	1	4	0	10	0	0	0	10	0	0	0
8. Cyst follicle	2	2	6	0	0	2	4	4	10	0	0	0
Average	2.5	1.5	4.8	1.3	4.1	2.4	2.3	1.3	8.1	1.9	0.0	0.0

The number of examined fields evaluated for each time point was 10. Grade of histopathological findings: – (no abnormality), + (slight), ++ (mild), +++ (moderate).

## 4. Conclusion

The current study aimed to investigate the repurposing approach to overcome fungus infection resistance besides the common side effects of conventional antifungal drugs. Atorvastatin as a repurposed drug was a good candidate due to its potential antimicrobial activity. Emulsome as vesicle nanosystem was selected to permeated ATO effectively to different skin layers. Several formulation parameters were optimized according to the OFAT design to reach the selected formula (E21) with low PS and PDI, and high ZP and EE%, Furthermore, the emulsome system controlled the drug release and encourage its penetration into skin strata which was inferred by CLSM studies. The *in vivo* irritation test confirmed the safety of the developed formulation. Both *in vitro* and *in vivo* microbiological studies showed promising antifungal activity of encapsulated atorvastatin which was similar to a commercial antifungal drug. Therefore, the developed formula could be used as an alternative therapeutic approach to current commercial medications for treating dermal fungal infections.
